# JNK Signaling Positively Regulates Acute Ethanol Tolerance in *C. elegans*

**DOI:** 10.3390/ijms25126398

**Published:** 2024-06-10

**Authors:** Changhoon Jee, Enkhzul Batsaikhan

**Affiliations:** Department of Pharmacology, Addiction Science and Toxicology, College of Medicine, University of Tennesse Health Science Center, Memphis, TN 38163, USA; ebatsaik@uthsc.edu

**Keywords:** ethanol vapor, locomotion, ethanol tolerance, *C. elegans*, JNK

## Abstract

Alcohol use disorder (AUD) is a chronic neurobehavioral condition characterized by a cycle of tolerance development, increased consumption, and reinstated craving and seeking behaviors during withdrawal. Understanding the intricate mechanisms of AUD necessitates reliable animal models reflecting its key features. *Caenorhabditis elegans* (*C. elegans*), with its conserved nervous system and genetic tractability, has emerged as a valuable model organism to study AUD. Here, we employ an ethanol vapor exposure model in *Caenorhabditis elegans*, recapitulating AUD features while maintaining high-throughput scalability. We demonstrate that ethanol vapor exposure induces intoxication-like behaviors, acute tolerance, and ethanol preference, akin to mammalian AUD traits. Leveraging this model, we elucidate the conserved role of c-jun N-terminal kinase (JNK) signaling in mediating acute ethanol tolerance. Mutants lacking JNK signaling components exhibit impaired tolerance development, highlighting JNK’s positive regulation. Furthermore, we detect ethanol-induced JNK activation in *C. elegans*. Our findings underscore the utility of *C. elegans* with ethanol vapor exposure for studying AUD and offer novel insights into the molecular mechanisms underlying acute ethanol tolerance through JNK signaling.

## 1. Introduction

Alcohol use disorder (AUD) is a growing public health concern, further exacerbated by the COVID-19 pandemic, resulting in increased alcohol consumption [1]. AUD is a complex brain disease with intricate, and thus incompletely understood, mechanisms, posing challenges for pharmacological interventions. Animal models that accurately replicate AUD’s key features are imperative for advancing our understanding and developing effective treatments. Chronic alcohol exposure induces a state of tolerance, prompting escalated intake to achieve comparable physiological effects [2,3,4] and consequently fostering escalated drinking patterns. Upon cessation of or a reduction in alcohol consumption, individuals grappling with AUD commonly experience withdrawal symptoms, marked by intense cravings and a compelling urge to resume drinking [5,6,7].

The invertebrate model, *Caenorhabditis elegans*, has emerged as a valuable model organism for studying alcohol-related behaviors, owing to its straightforward yet evolutionarily conserved nervous system, genetic manipulability, and amenability to high-throughput (HTP) screening [8,9,10,11,12,13,14,15]. Notably, the physiological responses of *C. elegans* to alcohol closely mirror those observed in humans, encompassing intoxication-like behavioral and physiological responses, acute functional tolerance, withdrawal symptoms, and ethanol preference [2,3,4,5,6,7,16]. Moreover, *C. elegans* can manifest an AUD-like dependent state, characterized by ethanol preference and aversive resistant seeking (ARS) [17,18]. Thus, in conjunction with its established attributes as a model organism [19,20,21,22,23], this model offers unique opportunities to explore the mechanisms underlying alcohol dependence. Previous studies investigating alcohol dependence in *C. elegans* have primarily relied on methodologies toward the use of solid or liquid media as a way to influence locomotion and internal concentration [8,14]. Despite successfully eliciting intoxication-like locomotion and ethanol-dependent behaviors such as preference and aversion-resistant ethanol seeking [9,17,18], the labor-intensive nature of these methods impedes the HTP capacity of this model. Hence, in this study, we sought to probe a *C. elegans* model of alcohol dependence using ethanol vapor exposure which emulates mammalian characteristics of AUD while preserving HTP capabilities. We systematically varied ethanol vapor concentration and exposure duration in *C. elegans* and scrutinized their behavioral and physiological responses.

A c-jun N-terminal kinase (JNK) signaling pathway, a key regulator of cellular stress responses, is increasingly recognized for its involvement in alcohol use disorder (AUD). Emerging evidence suggests a conserved role for JNK across species in mediating the acute behavioral effects of ethanol exposure [24]. Furthermore, genome-wide association studies (GWASs) in humans consistently associate MAPKKK9, a JNK pathway component, with susceptibility to AUD. Consequently, we investigated the conserved role of JNK signaling in alcohol-related phenotypes and demonstrated its significant contribution to the development of acute functional tolerance to ethanol using both our ethanol vapor approach and conventional methods. In sum, ethanol vapor exposure in *C. elegans* presents a promising model for elucidating AUD-related behaviors and molecular pathways.

## 2. Results

### 2.1. Ethanol Vapor Exposure Elicits Intoxication-like Behavioral Responses in C. elegans

**Figure 1:**
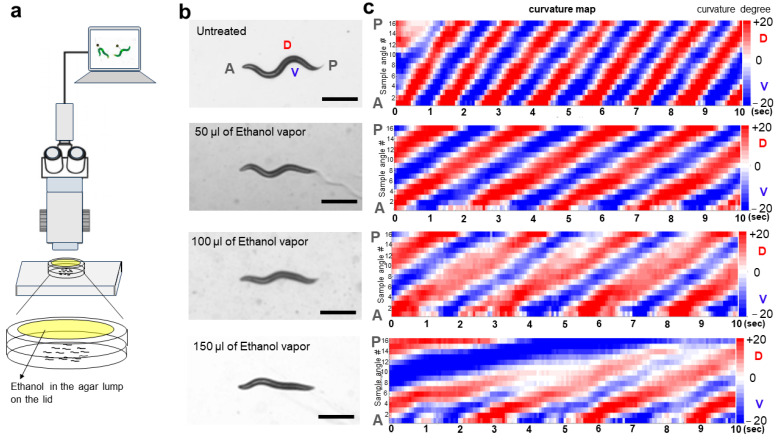
Behavioral response of wild-type *C. elegans* to ethanol vapor. (**a**) Schematic representation of ethanol vapor exposure setup for *C. elegans*. (**b**) Ethanol vapor-induced alterations in amplitude of body bends. Scale bar, 500 μm. (**c**) Example curvature map representing locomotion of worm moving forward under different concentrations of ethanol vapor. Red (**top** of scale) represents 20-degree angle and blue (**bottom** of scale) represents minus 20-degree angle. A, anterior of worms; P, posterior of worms; D, dorsal side of worms; V, ventral side of worms; sample angles, angles along worm’s centerline.

**Figure 2:**
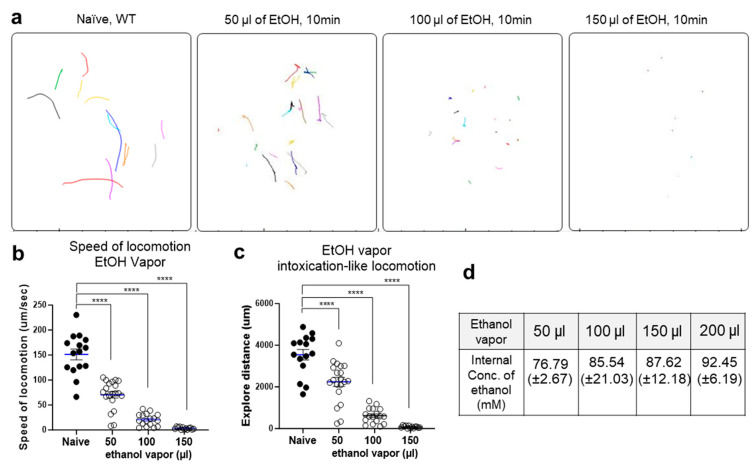

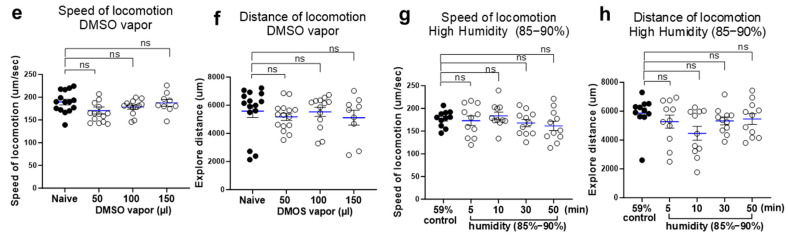
Intoxication-like locomotion of wild-type *C. elegans* after exposure to ethanol vapor. 50 µL, 100 µL, or 150 µL of ethanol was vaporized for 10 min, and similar behavioral effects indicative of intoxication were observed in a dosage-dependent manner. (**a**) The trajectory of individual naïve worms and those exposed to 50 µL, 100 µL, or 150 µL of ethanol vapor was analyzed. Worms exposed to ethanol vapor for 10 min were assessed for speed (**b**) and exploration distance (**c**). A one-way ANOVA comparison revealed significant differences in both speed [F(3, 61) = 94.25, *p* < 0.0001] and exploration distance [F(3, 65) = 64.21, *p* < 0.0001]. Significant post hoc differences (Dunnett’s test) between naïve and ethanol vapor-exposed WT animals across multiple concentrations are denoted (*p* < 0.0001, ****). The values are mean ± SEM. (**d**) The internal concentration of ethanol (mM) during 10 min of ethanol vaporization. (**e**,**f**) 50 µL, 100 µL, or 150 µL of DMSO was vaporized for 10 min. N ≥ 9 in each group. (e) Speed against DMSO vapor [F(3, 48) = 1.840, ns, *p* = 0.1526]; non-significant post hoc differences (Dunnett’s test) between naïve and DMSO vapor-exposed worms. (**f**) Exploration distance against DMSO vapor [F(3, 48) = 0.3790, ns, *p* = 0.7685]; non-significant post hoc differences (Dunnett’s test) between naïve and DMSO vapor-exposed worms. (**g**,**h**) WT animals were exposed to a higher humidity (85–90%) compared to room-ambient humidity (59%). N = 11 in each group. (**g**) Speed [F(4, 50) = 0.9789, ns, *p* = 0.4276]; non-significant post hoc differences (Dunnett’s test) between 59% and higher humidity. (**h**) Exploration [F(4, 50) = 1.691, ns, *p* = 0.1668]; non-significant post hoc differences (Dunnett’s test) between 59% and higher humidity. The black solid-colored circle represents the control group (mean ± SEM) to which the mean of each column (open-circle) was compared using Dunnett’s post hoc multiple comparison test. The blue line shows the mean and standard error of the mean (SEM).

The behaviors of *C. elegans* are significantly impacted by exposure to ethanol. The application of exogenous ethanol results in reversible, dose-dependent reductions in body bend amplitude, locomotion speed, and egg laying behavior [8,25]. We systematically varied the concentration of ethanol vapor among multiple groups of wild-type *C. elegans* and noted distinct changes in behavior. Worms were exposed to 50 µL, 100 µL, or 150 µL of ethanol vapor for 10 min. To maintain consistency with previous experiments [8,10,14,26], locomotion was recorded and assessed during the initial 10 min of ethanol vapor exposure. Notably, we observed dose-dependent alterations in body bend amplitude (Figure 1). These alterations were quantified through locomotion analysis using a curvature map, quantifying, and visualizing the movement patterns of *C. elegans*. The locomotion of *C. elegans* is characterized by an S-shaped posture due to continuous crawling on its side, with waves of dorsal-ventral (DV) body flexions propagating along the anterior-posterior axis. During *C. elegans* crawling, a full dorsoventral (DV) bending cycle is completed by the head before the bend propagates along the body from head to tail, with a dominant bending frequency of approximately 0.8 Hz [27]. Our curvature matrix representations revealed slightly slower frequency, amplitude, and propagation of bends in ethanol-exposed animals compared to untreated controls (Figure 1b,c). The curvature amplitude is color-coded and plotted along the animal’s centerline with the horizontal axis (time) and the vertical axis (position). Additionally, we conducted further locomotion analysis following acute exposure to ethanol vapor, evaluating the speed and exploration distance in the worms. Trajectories of individual naïve worms and those exposed to different concentrations of ethanol vapor were analyzed, revealing a dose-dependent reduction in locomotion speed and exploration distance (Figure 2). Compared to naïve worms, those exposed to different ethanol vapor concentrations (50 µL, 100 µL, or 150 µL) exhibited a significant reduction in both locomotion speed and exploration distance.

To correlate behavioral changes with internal ethanol levels, we assessed the internal ethanol levels in wild-type worms following 10 min of exposure to each vapor concentration. In a prior investigation employing solid NGM media for ethanol exposure, it was noted that internal tissue ethanol concentrations ranged from 70 to 90 mM in wild-type N2 worms exposed to 500 mM exogenous ethanol for over 10 min [26]. This internal concentration translates to a blood alcohol concentration (BAC) equivalent to approximately 0.32 to 0.41% in humans, which is known to induce significant intoxication [26]. Employing the same validated method, we quantified the internal ethanol concentration in wild-type nematodes following each vapor exposure session. The internal concentrations of ethanol were as follows: 50 µL of EtOH: 76.79 (±2.67), 100 µL of EtOH: 85.54 (±21.03), 150 µL of EtOH: 87.62 (±12.18), and 200 µL of EtOH: 92.45 (±6.19), as shown in Figure 2d. Our findings on internal ethanol concentration following vapor exposure are consistent with previous investigations utilizing solid NGM media for ethanol exposure.

Ethanol readily absorbs moisture from the air (hygroscopic) and mixes completely with water (miscible). To address this and investigate if ethanol’s interaction with water can affect worm locomotion experiments, we performed a control experiment using hygroscopic dimethyl sulfoxide (DMSO) at varying concentrations (50 µL, 100 µL, 150 µL), the same amount as that of the ethanol vapor (Figure 2a–c). We observed no significant changes in worm speed or travel distance with DMSO (Figure 2e,f). Additionally, exposing WT worms to a higher humidity (85–90%) did not affect their movement (Figure 2g,h). These results suggest that ethanol’s hygroscopic nature likely does not influence the observed locomotion changes.

### 2.2. Prolonged Exposure to Ethanol Vapor Induces Ethanol Tolerance and Ethanol Preference

**Figure 3:**
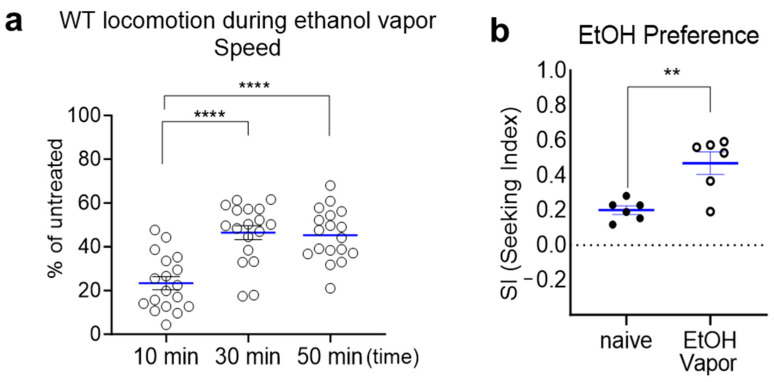
Prolonged exposure to ethanol vapor induces acute tolerance and ethanol-seeking behavior in *C. elegans*. (**a**) Development of acute tolerance to ethanol-induced depression of locomotion. Wild-type *C. elegans* were exposed to ethanol vapor for varying durations (10, 30, or 50 min). Locomotion behavior was assessed after each exposure period. One-way ANOVA revealed significant differences in locomotion depression across different time points (*p* < 0.0001, F(2, 51) = 19). Post hoc Tukey’s multiple comparison test indicated significant differences in locomotion between 10 min and 30 min exposure groups (****, *p* < 0.0001) as well as between 10 min and 50 min exposure groups (****, *p* < 0.0001). (**b**) Development of ethanol preference through prolonged ethanol vapor exposure. Wild-type *C. elegans* were exposed to ethanol vapor for 4 h, and chemotaxis to ethanol was assessed subsequently. Significant difference was observed by using Mann–Whitney test (**, *p* < 0.01). Values are represented as mean ± SEM (blue line). The open circle represents ethanol vapored groups.

The behavioral manifestations of wild-type worms in response to ethanol parallel the progression of AUD cycles observed in mammals, marked by the development of functional ethanol tolerance and an inclination toward ethanol preference [9,10,14,17,18]. In this context, we aimed to determine whether ethanol vapor induces functional ethanol tolerance and ethanol preference in wild-type *C. elegans*. Acute exposure to ethanol vapor induces a state of locomotor depression in *C. elegans*. Upon 10 min of exposure to ethanol vapor (100 µL), the locomotor speed of wild-type animals decreased to 23.31% ± 2.97% of that observed in untreated animals. However, the speed of wild-type animals exposed to ethanol vapor gradually increased over the course of vapor exposure, reaching 46.38% ± 3.19% at 30 min and 45.29% ± 2.79% at 50 min (Figure 3a). These findings indicate that *C. elegans* develops acute tolerance to the depressive effects of ethanol vapor, akin to those observed with ethanol intake via solid or liquid media. Furthermore, prolonged exposure to ethanol vapor also elicited experience-dependent preference and seeking behavior in *C. elegans* (Figure 3b). Remarkably, *C. elegans* exhibits proactive ethanol-seeking behavior, somewhat resembling alcohol self-administration in mammals. Indeed, ethanol preference developed through pretreatment enabled WT animals to traverse a distance approximately 100 times their own body size (<1 mm) to navigate toward the ethanol spot. The seeking index (SI) was obtained as represented in previous studies [17,28]. A high SI indicates that the ethanol acts as a strong attractant, which corresponds to the development of preference through experiencing ethanol exposure. These findings further corroborate and extend observations from previous investigations into ethanol-related behaviors in *C. elegans*, demonstrating that vapor exposure can effectively induce AUD-like phenotypes in this model organism.

### 2.3. JNK Signaling Cascades Positively Regulate Acute Ethanol Tolerance

Mitogen-activated protein kinases (MAPKs) are intricately involved in stress response mechanisms, orchestrating transcriptional alterations, cellular morphology adjustments, differentiation processes, proliferation dynamics, and even programmed cell death [29,30]. Ethanol is known for its regulatory influence on MAPK signaling cascades across various tissues, spanning from invertebrates to mammals, with particular emphasis on the complex neural networks within the brain [24,31,32,33]. In particular, signaling pathways involving c-Jun N-terminal kinase (JNK) have been shown as potential mediators of alcohol-induced stress responses, with evidence for a conserved role in flies and mice in regulating sensitivity to the acute behavioral effects of ethanol exposure [34]. Furthermore, substantial genome-wide association studies (GWASs) in humans have consistently identified MAPKKK9, involved in the JNK signaling pathway, as a potential risk gene associated with alcohol dependence [35]. We substantially, asked whether the ethanol vapor approach could be a viable and useful tool for the assessment of biologically significant pathways associated with AUD. To address this question, we utilized knockout (KO) animals lacking *jnk-1*, investigating its involvement in the development of acute tolerance to ethanol exposure. The human genome includes three genes that encode JNK proteins, JNK1 (*MAPK8*; ENSG00000107643), JNK2 (*MAPK9*; ENSG00000050748), and JNK3 (*MAPK10*; ENSG00000109339) [36,37]. *Jnk-1* has been predicted as an orthologue of JNKs in *C. elegans* [38] and their conserved roles are functionally validated [39]. Accordingly, we tested knockout (KO) animals with *jnk-1* to determine the development of acute tolerance to ethanol vapor.

**Figure 4:**
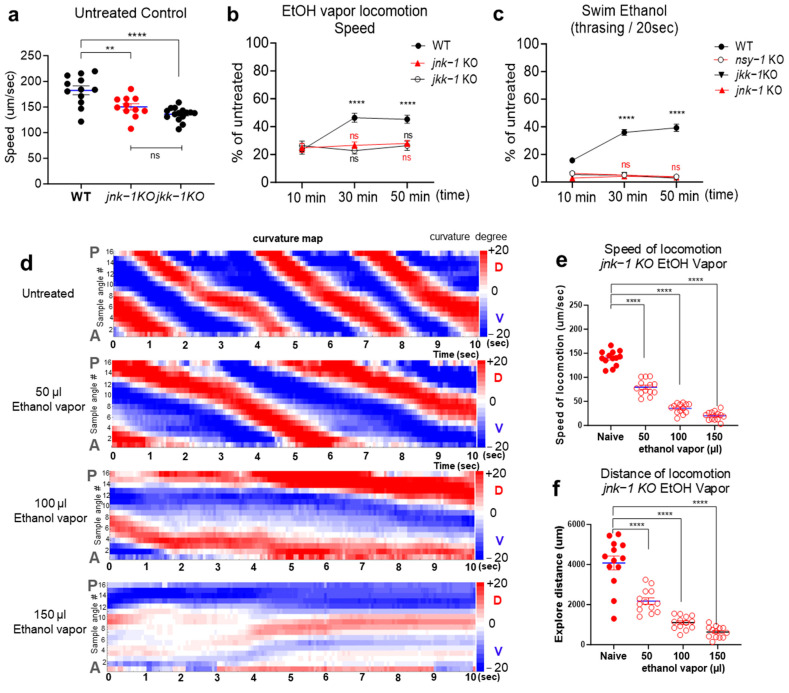
JNK signaling facilitates the development of ethanol tolerance. (**a**) The control speed of naive animals. A one-way ANOVA (*p* < 0.0001, F(2, 35) = 15.89) revealed a slight reduction in locomotion speed compared to wild-type animals; however, mutations in *jnk-1*(*gv7*) and *jkk-1*(*km2*) do not impair the overall ability of locomotion. A post hoc Tukey’s multiple comparison test showed significant differences in locomotion between WT and *jnk-1*(*gv7*) animals (red, **, *p* < 0.01) and between WT and *jkk-1*(*km2*) animals (****, *p* < 0.0001). (**b**) The development of ethanol tolerance through prolonged ethanol vapor (100 µL). A two-way ANOVA comparison shows the impaired development in *jnk-1*(*gv7*) and *jkk-1*(*km2*) animals [F_Genotype_(2, 186) = 17.17, *p* < 0.0001; F_time_(2, 186) = 7.172, *p* < 0.001; F_Genotype x time_(4, 186) = 6.259, *p* < 0.0001]. A significant post hoc differences Tukey’s multiple comparison between the 10 min and 30 min/50 min in each genotype [*p* < 0.0001, **** for WT; *p* > 0.05, ns for *jnk-1*(*gv7*) and *jkk-1*(*km2*)]. The values are mean ± SEM. N ≥ 18 in each genotype across all time points. (**c**) The development of ethanol tolerance during swimming in liquid environments containing ethanol (400 mM M9). A two-way ANOVA comparison shows the impaired development in *nsy-1*(*ok593*), *jnk-1*(*gv7*), and *jkk-1*(*km2*) animals [F_Genotype_(3, 336) = 265.7, *p* < 0.0001; F_time_(2, 336) = 16.16, *p* < 0.001; F_Genotype x time_(6, 336) = 23.33, *p* < 0.0001]. A significant post hoc differences Tukey’s multiple comparison between the 10 min and 30 min/50 min in each genotype [*p* < 0.0001, **** for WT; *p* > 0.05, ns for *nsy-1*(*ok593*), *jnk-1*(*gv7*), and *jkk-1*(*km2*)]. The values are mean ± SEM. N ≥ 26 in each genotype across all time points. (**d**) An example curvature map representing the locomotion of a *jnk-1*(*gv7*) worm moving forward under different concentrations of ethanol vapor. Red (**top** of the scale) represents a 20-degree angle and blue (**bottom** of the scale) represents a minus 20-degree angle. A, anterior of worms; P, posterior of worms; D, dorsal side of worms; V, ventral side of worms; sample angles, angles along the worm’s centerline. (**e**,**f**) *jnk-1*(*gv7*) animals exposed to ethanol vapor for 10 min were assessed for speed (**e**) and exploration distance (**f**). The solid-colored circle represents the control group (mean ± SEM) to which the mean of each column (open-circle) was compared using Dunnett’s post hoc multiple comparison test. A one-way ANOVA comparison revealed significant differences in both speed [F(3, 48) = 220.4, *p* < 0.0001] (**e**) and exploration distance [F(3, 48) = 59.08, *p* < 0.0001] (**f**). A significant post hoc difference test (Dunnett’s test) between naïve and ethanol vapor-exposed WT animals across multiple concentrations are denoted (*p* < 0.0001, ****). The values are mean ± SEM (blue line). N = 13 in each group.

JNK-1 is recognized for its regulatory role in various facets of *C. elegans* physiology and behavior, particularly in modulating altered locomotion patterns, diminished exploratory behavior, and compromised responses to stressors [39,40]. Previous studies have shown that *jnk-1*(*gk7*) mutants exhibit defective body movement coordination, characterized by loopy and exaggerated movements, which are distinct from the uncoordinated locomotion observed in “unc” mutants [39]. These loopy and exaggerated body bends result in a substantial reduction in the distance covered during the recorded time, leading to a slight reduction in locomotion speed compared to wild-type animals (Figure 4a). However, the mutation in *jnk-1*(*gv7*) does not impair overall locomotive ability. Our ethanol vapor assay, conducted on an unseeded NGM plate without bacteria (food), demonstrates the constant stimulated locomotion of naive *jnk-1*(*gk7*) mutants, as shown in Figure 4d–f. Building upon the findings in Figure 2, 100 µL ethanol vapor exposure was selected, and accordingly, during ethanol vaporization, locomotion was recorded at 10 min, 30 min, and 50 min time points. We observed impaired development of acute tolerance to ethanol in *jnk-1*(*gv7*) mutants compared with WT animals (Figure 4b). Compared with *jnk-1*(*gv7*), WT animals recovered more rapidly from the depressive effect ethanol vaporization on locomotion. To further elucidate the role of this signaling pathway, we investigated *jkk-1*, the upstream MAPKK7 (ENSG00000076984) orthologue [38] known to regulate *jnk-1* in *C. elegans* body movement and heavy metal stress response [39]. Biochemical approaches also showed that JKK-1 was a specific activator of JNK-1 [40]. Similar to *jnk-1*(*gv7*) mutants, *jkk-1* KO animals displayed a slight reduction in locomotion speed due to their distinct locomotor patterns (Figure 4a). Notably, KO animals with *jkk-1* reproduced the impaired development of acute tolerance to ethanol vapor observed in *jnk-1* animals (Figure 4b). These results suggest that JNK signaling positively regulates the development of acute tolerance to ethanol in *C. elegans*.

Further assessment of the pathway was conducted employing the robust swimming assay in liquid environments supplemented with ethanol. Previous investigations have underscored the utility of this assay in NGM or M9 media containing ethanol, attributing its effectiveness to the stark distinction between crawling and swimming behaviors [27]. This distinction facilitates the robust assessment of acute tolerance development to ethanol [10]. Therefore, we expanded our investigation to include *jnk-1*KO and *jkk-1*KO mutants in the ethanol swimming assay, alongside *nsy-1*, the upstream MAPKKK5/ASK1 (ENSG00000197442) orthologue [38] known to activate JNK and p38 MAPKs [41,42,43]. Exposure to 400 mM ethanol resulted in the suppression of swimming behavior, inducing intoxication-like depressive swimming in all tested groups. Notably, unlike WT animals, KO animals with *nsy-1*, *jkk-1*, and *jnk-1* exhibited a deficit in recovery from the ethanol-induced depressive effect, suggesting a potential impairment in the development of acute tolerance to ethanol (Figure 4c). Compared to naïve *jnk-1*(*gv7*) animals, those exposed to different ethanol vapor concentrations (50 µL, 100 µL, or 150 µL) exhibited a significant reduction in both locomotion speed and exploration distance (Figure 4d–f).

### 2.4. Activation of JNK-1 upon Exposure to Ethanol

**Figure 5:**
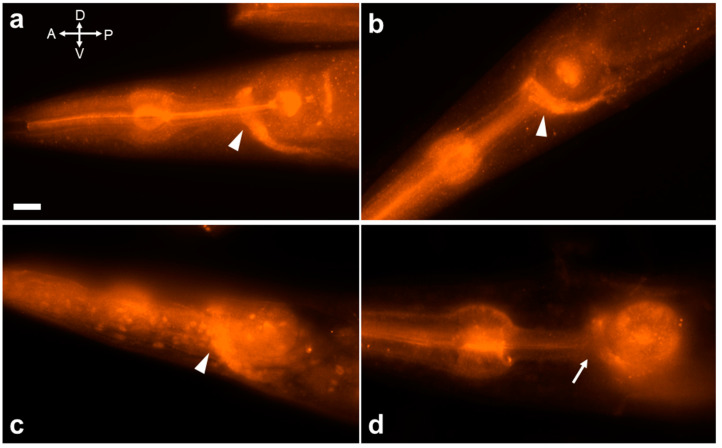
JNK-1 activation in the *C. elegans* nervous system, primarily in the nerve ring, following exposure to ethanol. (**a**–**c**). Wild-type *C. elegans* worms exposed to various stimuli: (**a**) 400 mM ethanol on a plate, (**b**) 37 °C heat shock stress, and (**c**) 400 mM ethanol in M9 buffer. The white arrowheads indicate the distinct expression of activated JNK-1 within the nerve ring. (**d**) A wild-type *C. elegans* worm in M9 buffer. The white arrow indicates the location of the nerve ring. The scale bar is 10 µm.

MAPK cascades are highly conserved across evolution. The evolutionarily conserved MAPK cascade relays environmental stress signals through sequential kinase phosphorylation [44,45,46]. Activated GPCRs trigger upstream MAPKKKs (like ASK1) to phosphorylate and activate downstream MAPKKs (like JKK-1). JKK-1 then phosphorylates JNKs, leading to their activation. We sought to determine if JNK-1 activation occurs during acute ethanol exposure using an anti-phospho-SAPK/JNK antibody. This antibody has been previously demonstrated to effectively detect JNK-1 activation in *C. elegans* [47]. Robust activation of JNK-1 was identified within the nerve ring and major nerve tracts, including the ventral nerve cord (VNC) and retrovesicular ganglia (RVG) (Figure 5a). Notably, this activation pattern mirrored that observed under heat shock stress conditions (Figure 5b), aligning with previous reports [47]. Furthermore, JNK-1 was strongly activated within the nerve ring and major nerve tracts during ethanol swimming conditions as well (Figure 5c). Interestingly, we observed JNK-1 nuclear translocation under these conditions. This finding aligns with previous reports where cells expressing JNK-1::GFP exhibited localization in both the cytoplasm and nucleus, while the JKK-1::GFP fusion protein was excluded from the nucleus [40]. Additionally, M9 control experiments validated our observations, showing little JNK-1 activation within the nerve ring, with only faint staining in the VNC and RVG (Figure 5d).

## 3. Discussion

Alcohol vapor is a widely used tool in animal models to induce AUD-related phenotypes from invertebrates to mammals, including eliciting an acute tolerance response in Drosophila and rodents [48,49]. Especially in rodent models, alcohol vapor inhalation stands as a frequently employed method, reliably inducing forced exposure and establishing physical alcohol dependence, thereby influencing subsequent alcohol consumption behavior [50,51]. This method offers meticulous control over dosage, duration, and temporal alcohol exposure patterns throughout the experimental procedure. Employing chronic vapor exposure with an intermittent access paradigm has proven effective in generating significantly elevated levels of alcohol self-administration, persisting long after acute withdrawal [52,53]. This prolonged exposure leads to increased alcohol consumption and results in enduring dysregulation of neuronal gene expression profiles, underscoring the profound and lasting impact of chronic alcohol vapor exposure [54]. Furthermore, chronic intermittent exposure to ethanol vapor has been demonstrated to promote binge-like drinking and drinking despite negative consequences, making it a reliable tool for studying aversion-resistant seeking of ethanol (ARS) [55].

We investigated the effects of ethanol vapor exposure on *C. elegans* and demonstrated that *C. elegans* exposed to ethanol vapor exhibits behavioral alterations reminiscent of AUD in mammals. As with conventional ethanol exposure methods in *C. elegans*, the dose-dependent decrease in locomotion speed and exploration distance observed upon ethanol vapor exposure aligns with intoxication-like behaviors. Moreover, the development of functional tolerance to the depressive effects of ethanol over time reflects a characteristic feature of AUD. Furthermore, here, we incorporated the ethanol vapor paradigm to distinguish ethanol-induced preference from potential confounding variables, such as caloric value. Additionally, we demonstrated that exposure to ethanol vapor elicited ethanol preference and seeking behavior in *C. elegans*, further strengthening the parallels with AUD. These findings suggest that ethanol vapor exposure offers a viable approach to model AUD-like behaviors in *C. elegans*. Our study further demonstrates the effectiveness of ethanol vapor exposure by facilitating the dissection of the underlying molecular mechanisms using genetic approaches. The identification of JNK signaling as a positive regulator of acute ethanol tolerance exemplifies this potential and highlights the promise of *C. elegans* with ethanol vapor exposure as a valuable tool for unraveling the complexities of AUD. 

Eukaryotic cells are universally equipped with the intricate network of MAPK signaling cascades, which exert a profound influence on a multitude of cellular processes, encompassing gene expression, mitosis, cell growth, migration, and survival [56,57]. In *C. elegans*, JNK signaling exhibits remarkable evolutionary conservation, impacting various cellular processes. It is activated by a spectrum of stresses and plays a crucial role in mediating cellular responses to maintain homeostasis [47,58,59]. *jnk-1* signaling has been implicated in the response to diverse stressors, including heavy metal stress [39,60], heat stress [47,61], and osmotic stress [59]. Beyond its role in stress response, the JNK signaling cascade in *C. elegans* plays a significant role in neuronal function [40,62]. Notably, it exhibits particular relevance in D-type GABAergic neurons, where JNK-1 and JKK-1 regulate coordinated movement. This aligns well with the established importance of the JNK cascade for physiological functions in neuronal cells in mammals [63]. 

We have demonstrated that JNK signaling positively regulates acute ethanol tolerance in worms. We investigated the role of the JNK signaling pathway in ethanol tolerance using *jnk-1* and *jkk-1* knockout mutants. Compared to wild-type animals, *jnk-1* and *jkk-1* mutants displayed impaired development of acute tolerance to ethanol vapor, as evidenced by their slower recovery from the ethanol-induced depressive effects on locomotion (Figure 4b). These observations were further corroborated in the swimming assay, where *nsy-1*, *jkk-1*, and *jnk-1* mutants all exhibited deficits in recovery from ethanol-induced depressive swimming behavior (Figure 4c). This finding suggests that the activation of MAPK cascades is required for the development of acute tolerance to ethanol in *C. elegans*. 

MAPK cascades are highly conserved across evolution. These operate through sequential phosphorylation and activation of specific kinases within the cascade, culminating in the phosphorylation of target regulatory proteins by both MAPK and MAPKAPK components [64]. In response to numerous stimuli, including environmental stress, activated GPCR signaling stimulates the phosphorylation and activation of several upstream MAPKKKs, such as ASK1. These activated MAPKKKs, in turn, phosphorylate and activate downstream MAPKKs (MKK4 and MKK7). Finally, the activated MAPKKs such as JKK-1 phosphorylate JNKs on threonine and tyrosine residues, leading to their activation [44,45,46]. In *C. elegans*, JNK-1 expression was initially characterized using translational green fluorescent protein (GFP) fusions, revealing its presence in most neurons throughout development [40]. Subsequent investigations employing whole-mount immunohistochemistry with an anti-phospho-SAPK/JNK antibody demonstrated stress-induced activation of JNK-1, specifically within the nerve ring near the pharynx. This activation is likely mediated by its upstream kinase, JKK-1 [47]. Building upon this established methodology, we demonstrated the activation of JNK-1 upon exposure to ethanol. We observed robust JNK-1 activation within the nerve ring and major nerve tracts, mirroring the pattern seen under heat stress. Notably, ethanol exposure in liquid environments also triggered JNK-1 nuclear translocation, aligning with previous reports on JNK-1 localization [40].

JNK activation is triggered by diverse stimuli, leading to its phosphorylation and subsequent nuclear translocation. JNK is implicated in the multisite phosphorylation of the c-JUN transactivation domain, a central component of the AP-1 (activator protein 1) transcription factor [65,66]. This phosphorylation event modulates c-JUN’s interaction with partner proteins, thereby regulating the expression of target genes crucial for cell proliferation and potentially contributing to disease pathophysiology [67]. AP-1 is composed of a heterodimeric complex formed by members of the JUN family, including c-JUN, and FOS proteins, including c-FOS [68,69,70]. AP-1 dimers, formed between *C. elegans jun-1* and *fos-1*, and their functional conservation have been reported in the reproductive system [71]. *C. elegans* expresses multiple MAPK signaling pathways with distinct tissue-specific distributions. Notably, *jnk-1* and its upstream activator *jkk-1* are primarily found in neuronal cells, while *kgb-1*, another JNK-like MAPK, exhibits broader expression across tissues like muscle, hypodermis, intestine, and germ cells [59]. The presence of CRE and ETS binding sites in *C. elegans jun-1* and *fos-1* promoters, respectively, suggests potential regulation by JNK signaling [59]. This hypothesis is further supported by studies showing KGB-1 dependence on *jun-1* expression [72] and JNK-1-mediated c-Elk1 phosphorylation [39], a known activator of c-Fos. Interestingly, different isoforms of the *Jun* and *Fos* genes seem to be expressed in distinct cell types of *C. elegans* [71], hinting at a potential role of JNK signaling tailored to specific tissues. While stimulus-induced JNK nuclear translocation is known to initiate and regulate physiological functions in mammals, it is especially importantly implicated in pathophysiological processes [73]. Substantially, we observed the exclusive observation of JNK nuclear translocation only in the liquid environment during ethanol exposure during forced swimming (Figure 5c), suggesting heightened cellular stress due to ethanol’s combination with lower oxygen levels in the liquid and vigorous movements of swimming.

In the adult mammalian brain, JNKs are most likely present in their activated form in the absence of specific stimulation and localized to neurites, potentially acting as retrograde signaling molecules, suggesting a potential role in neuronal plasticity and memory formation [74,75,76]. In mammals, repeated exposure to ethanol leads to ethanol dependence and associated dysregulation of JNK phosphorylation in the central nervous system. Such dysregulation is evident within the hippocampus, frontal cortical regions, and striatum of adult male Wistar rats [77]. Subsequent investigations demonstrated that a decreased level of phosphorylated JNK was observed with chronic alcohol exposure during withdrawal. Nevertheless, the contribution of reduced JNK activation during withdrawal to escalated alcohol consumption and the promotion of dependence remains an area ripe for further exploration. Meta-analyses of human genome-wide association studies (GWASs) have suggested significant associations between JNK (MAPKKK9) loci and alcohol dependence [35], indicating a potential role of JNK signaling in the progression of alcohol dependence during chronic exposure. Future research should delve into exploring the involvement of JNK signaling in both the initiation and perpetuation of chronic ethanol dependence. Leveraging the ethanol vapor paradigm established in *C. elegans*, our study provides a promising avenue for comprehensive investigations into alcohol dependence progression during chronic exposure and withdrawal. This could involve implementing chronic ethanol vapor exposure protocols and evaluating withdrawal manifestations, ethanol preference [9,17], resistance to aversive ethanol-seeking behaviors [18], and patterns of JNK activation in both wild-type and JNK mutant worms. Moreover, experiments involving the overexpression of JNK-1 and cell-specific rescue approaches could provide valuable insights into the mechanisms underlying alcohol use disorder (AUD)-related behaviors and the contribution of evolutionarily conserved JNK signaling pathways to the development and perpetuation of alcohol dependence. Overall, our findings underscore the significance of ethanol vapor exposure as a tool for studying AUD-related behaviors in *C. elegans* and offer novel insights into the molecular mechanisms underlying acute ethanol tolerance mediated by JNK signaling, contributing to our understanding of alcohol dependence.

## 4. Materials and Methods

### 4.1. Strains and Maintenance

All strains were cultivated on nematode growth medium (NGM) plates with *Escherichia coli* (OP50) at 20 °C as described in [78], and the hermaphrodite worm was used for behavioral analysis. The Bristol N2 strain was used as wild-type (WT) animals. The strains *nsy-1*(*ok593*), *jkk-1*(*km2*), and *jnk-1*(*gv7*) were obtained from Caenorhabditis Genetics Center (CGC, Minneapolis, MN, USA), which is supported by the National Institutes of Health Office of Research Infrastructure Programs (P40 OD010440). 

### 4.2. Locomotion Analysis and Ethanol Vapor 

The unseeded NGM plates were dried for 2 h at 30 °C to ensure a stable surface for the copper rings. The copper rings were then carefully melted into the center of the solidified agar surface. Fifteen 1-day-old adult worms were transferred to the center of each copper ring on the NGM plate. A total of 1.5 mL of 2% agar was added in the middle of the plate lid for the agar plug. Ice-cold (at 4 °C) ethanol (200 proof) was added systematically in varying concentrations (50 µL, 100 µL, or 150 µL) directly onto the agar plug in the center of the plate lid. The plates were then sealed with Parafilm to trap the ethanol vapor. Following ethanol vaporization, locomotion was recorded using WormLab software (MBF Bioscience, Williston, VT, USA). The trajectory, speed, and travel distance were analyzed by using Wormlab software (MBF Bioscience, USA). To address the potential influence of ethanol’s hygroscopic nature on worm locomotion, additional control experiments were performed. Unseeded NGM plates were prepared as described above. Hygroscopic dimethyl sulfoxide (DMSO) at varying concentrations (50 µL, 100 µL, or 150 µL) was used to mimic the same amount of ethanol vapor exposure. Worm locomotion was recorded, and trajectory, speed, and travel distance were analyzed as described above. WT *C. elegans* were exposed to a higher humidity (85–90%) for varying time periods (5 min, 10 min, 30 min, and 50 min) in a customized humid chamber. Water was vaporized using an ultrasonic humidifier, and humidity was monitored with a humidity monitor (SC42, Smartro, Seattle, WA, USA). Worm locomotion was recorded at higher humidity levels (85% for 5 and 10 min, 90% for 30 and 50 min), and trajectory, speed, and travel distance were analyzed by using WormLab software (MBF Bioscience, USA).

### 4.3. Curvature Map Analysis 

Wild-type *C. elegans* were exposed to varying concentrations of ethanol vapor for 10 min. Locomotion was assessed by analyzing the amplitude of body bends, a measure of active sinusoidal movement. The curvature map was analyzed as previously described in [27].

### 4.4. Measurement of Internal Ethanol Concentration Using Spectrophotometric Analysis 

Several hundred 1-day-old adult worms were placed on unseeded plates containing the appropriate amount of ethanol vapor (described in locomotion assays) for the designated time. Internal concentrations of ethanol were followed as described in a previous study [26]. A total of 200 worms were transferred from the plates to a tube containing 15 μL ddH_2_O. Picking the 200 worms took an average of 2 min per condition. The tubes were placed at −80 °C until analysis. The worms were thawed on ice and ground in the tube with a pestle (BioMasherII tissue homogenizer, DWK Life Sciences Kimble™). The worm homogenate was stored at −20 °C. *C. elegans* homogenates were tested for ethanol concentration according to the manufacturer’s directions using an Alcohol Reagent Kit (Pointe Scientific, Canton, MI, USA). A total of 1.5 μL of worm homogenate was added to 300 μL of ice-cold alcohol reagent and incubated for five minutes at 30 °C, and the reaction was stopped by putting the tube into an ice-cold block. Ethanol concentration was determined by measuring the absorbance at 340 nm.

### 4.5. Ethanol Tolerance Assay 

Worms were exposed to ethanol vapor (100 µL) as described above and locomotion was recorded at 10 min, 30 min, and 50 min time points during ethanol vaporization. The trajectory, speed, and travel distance were analyzed by using WormLab software (MBF Bioscience). The ethanol swimming assay was adapted from a previously described method [10]. Briefly, the assay plate (1.6% BBL-Agar, 5 mM potassium phosphate, pH 6.0, 1 mM CaCl_2_, and 1 mM MgSO_4_) was prepared on the day of assay, and ethanol was added to reach a concentration of 400 mM. Then, M9 buffer solution containing ethanol (400 mM) was added to the assay plate and 15 well-fed 1-day-old adult animals were transferred. We obtained similar results using an NGM buffer. Swimming on ethanol or M9 (for untreated control) was recorded for 1 min (20 frames/second) at 10, 30, and 50 min. The number of body bends per 20 s were analyzed to calculate the values for relative thrashing (treated/untreated × 100). Statistical analysis was performed using a two-way ANOVA with Tukey’s multiple comparison post hoc test.

### 4.6. Ethanol Preference Assay 

One-day-old WT adult worms were exposed to ethanol vapor (100 µL) for 4 h. The chemotaxis to ethanol assay was performed and the seeking index was obtained, as previously described [17,18]. Briefly, chemotaxis plates were prepared with 10 cm Petri dishes that contained 10 mL of assay agar (2% agar, 5 mM of KPO_4_ [pH6], 1 mM of CaCl_2_, 1 mM of MgSO_4_). The worms were washed twice in S-basal and once in distilled water and placed onto spots on the plates.

### 4.7. Statistical Analysis 

The mean and standard error of the mean (SEM) were determined for all experimental parameters. The data were analyzed employing the two-way ANOVA test (Graph pad prism version 8.0.1). Values below 0.05 were considered as significant.

### 4.8. Immunochemistry for Phosphorylated JNK-1 

Whole-mount antibody staining was performed as previously described [47,79]. Briefly, 1:100 diluted rabbit anti-phospho-SAPK/JNK (Thr183/Tyr185) antibody (#4668; Cell Signaling Technology, Inc., Danvers, MA, USA) was used as the primary antibody to stain each group of worms (400 mM ethanol on plate, 37 °C heat stress, 400 mM ethanol swimming, and M9 swimming). This antibody recognizes a highly conserved protein motif in human JNK, which is also present in *C. elegans* JNK-1α/β, as described in [47]. For the cohort of worms exposed to 400 mM ethanol on plates, one-day-old adult wild-type (WT) worms were subjected to 400 mM ethanol treatment on NGM plates for 30 min. Prior to ethanol exposure, a half-region of each NGM plate was seeded with OP50 bacteria one day in advance. Subsequently, ethanol was applied to the unseeded half of the plate and allowed to diffuse for 2 h, resulting in a final concentration of 400 mM. The plates were then sealed with Parafilm to prevent ethanol evaporation. Following diffusion, WT worms were transferred to the plate containing both OP50 and 400 mM ethanol and incubated for 30 min before being harvested for staining. For the cohort subjected to heat stress, one-day-old adult WT worms were exposed to a temperature of 37 °C (Precision Gravity-Convection Incubator, #Cat. 51221085) for 1 h prior to harvest for staining. For the cohort undergoing ethanol swimming, one-day-old adult WT worms were allowed to swim in M9 buffer containing 400 mM ethanol for 30 min before being harvested for staining. As for the control group, one-day-old adult WT worms were permitted to swim in M9 buffer for 30 min before being harvested for staining.

## Data Availability

Data are contained within the article.
